# Nutritional status modifies pregnane X receptor regulated transcriptome

**DOI:** 10.1038/s41598-019-53101-9

**Published:** 2019-11-13

**Authors:** Fatemeh Hassani-Nezhad-Gashti, Outi Kummu, Mikko Karpale, Jaana Rysä, Jukka Hakkola

**Affiliations:** 1 Research Unit of Biomedicine, Pharmacology and Toxicology, University of Oulu, Oulu, Finland; 2 Medical Research Center Oulu, Oulu University Hospital and University of Oulu, Oulu, Finland; 3 School of Pharmacy, Faculty of Health Sciences, University of Eastern Finland, Kuopio, Finland

**Keywords:** Transcriptomics, Homeostasis, Hepatocytes

## Abstract

Pregnane X receptor (PXR) regulates glucose and lipid metabolism, but little is known of the nutritional regulation of PXR function. We investigated the genome wide effects of the nutritional status on the PXR mediated gene regulation in the liver. Mice were treated with a PXR ligand pregnenolone 16α-carbonitrile (PCN) for 4 days and subsequently either fasted for 5 hours or after 4-hour fast treated with intragastric glucose 1 hour before sample collection. Gene expression microarray study indicated that PCN both induced and repressed much higher number of genes in the glucose fed mice and the induction of multiple well-established PXR target genes was potentiated by glucose. A subset of genes, including bile acid synthesis gene *Cyp8b1*, responded in an opposite direction during fasting and after glucose feeding. PXR knockout abolished these effects. In agreement with the *Cyp8b1* regulation, PCN also modified the bile acid composition in the glucose fed mice. Contribution of glucose, insulin and glucagon on the observed nutritional effects was investigated in primary hepatocytes. However, only mild impact on PXR function was observed. These results show that nutritional status modifies the PXR regulated transcriptome both qualitatively and quantitatively and reveal a complex crosstalk between PXR and energy homeostasis.

## Introduction

Pregnane X receptor (PXR) is a master xenobiotic-sensing nuclear receptor. PXR has a broad ligand preference and it accepts numerous clinically used drugs and environmental contaminants as ligands^1^. Also, endogenous ligands such as some bile acids can activate PXR. PXR was originally identified to regulate drug metabolism and detoxification, i.e., functions logically connected with the sensing of chemical environment^2^. Later, PXR has been shown to regulate a number of other important cellular functions such as glucose, lipid and bile acid metabolism, inflammation, cell cycle and apoptosis^3,4^.

PXR affects multiple aspects of hepatic energy metabolism^3^. PXR activation was first shown to downregulate key genes involved in gluconeogenesis^5,6^. Recently, we reported that PXR activation impairs postprandial glucose tolerance and dysregulates expression and function of key glucose utilization factors such as glucose transporter 2 (GLUT2)^7,8^. Moreover, PXR activation induces synthesis and uptake of fatty acids and liver steatosis^3,9,10^. In fact, PXR knockout protects from high-fat diet induced obesity, liver steatosis and insulin resistance^10^.

Relatively little is known of how energy homeostasis regulates PXR function^3^. PXR expression is modestly induced in rat liver after 24 to 48 hours fasting, possibly involving peroxisome proliferator-activated receptor gamma coactivator 1-alpha (PGC-1α)^11^. PXR also interacts with multiple transcription factors, such as forkhead box protein O1 (FOXO1) and sterol regulatory element-binding protein 1 (SREBP1), which are heavily regulated by energy homeostasis^6,12^. Furthermore, a cellular energy sensor sirtuin 1 interacts with and deacetylates PXR^11,13^, which may affect PXR dimerization and DNA binding^14^. Low glucose has been described to induce PXR Ser^350^ phosphorylation in cell models^15^. Furthermore, fasting induced this phosphorylation in the mouse liver^15^. These observations suggest that PXR function and target gene regulation could be controlled by nutritional status and cellular energy state. However, this has not been studied systematically.

In the current study, we investigated genome wide effects of nutritional status on PXR function in the mouse liver *in vivo* by utilizing glucose feeding as a model. We show that compared to fasting, glucose feeding powerfully modifies the PXR target gene response.

## Results

### Nutritional status modifies PCN regulated transcriptome

We hypothesized that nutritional status may modify PXR function and the regulation of its target genes. To test this hypothesis, we performed an animal experiment investigating effect of fasting and glucose feeding on the PXR regulated transcriptome in liver. The study was designed to investigate the immediate effects of fasting to feeding transition on transcriptome under the control of PXR. The mice were first treated with a well-established PXR ligand PCN (50 mg/kg, i.p.), or with vehicle, for four days after which they were either fasted for 5 hours before sacrificing or were first fasted for 4 hours and were then given glucose (2 g/kg) by oral gavage 1 h before sacrificing. The glucose dose is similar to the one we have been using previously in the oral glucose tolerance test and the 1-hour time point for the samples collection after the glucose dosing was selected because in the oral glucose tolerance test the plasma glucose level typically remains elevated for about 60 minutes^7^. The liver samples were collected and the differentially expressed genes were analyzed with gene expression arrays.

**Figure 1:**
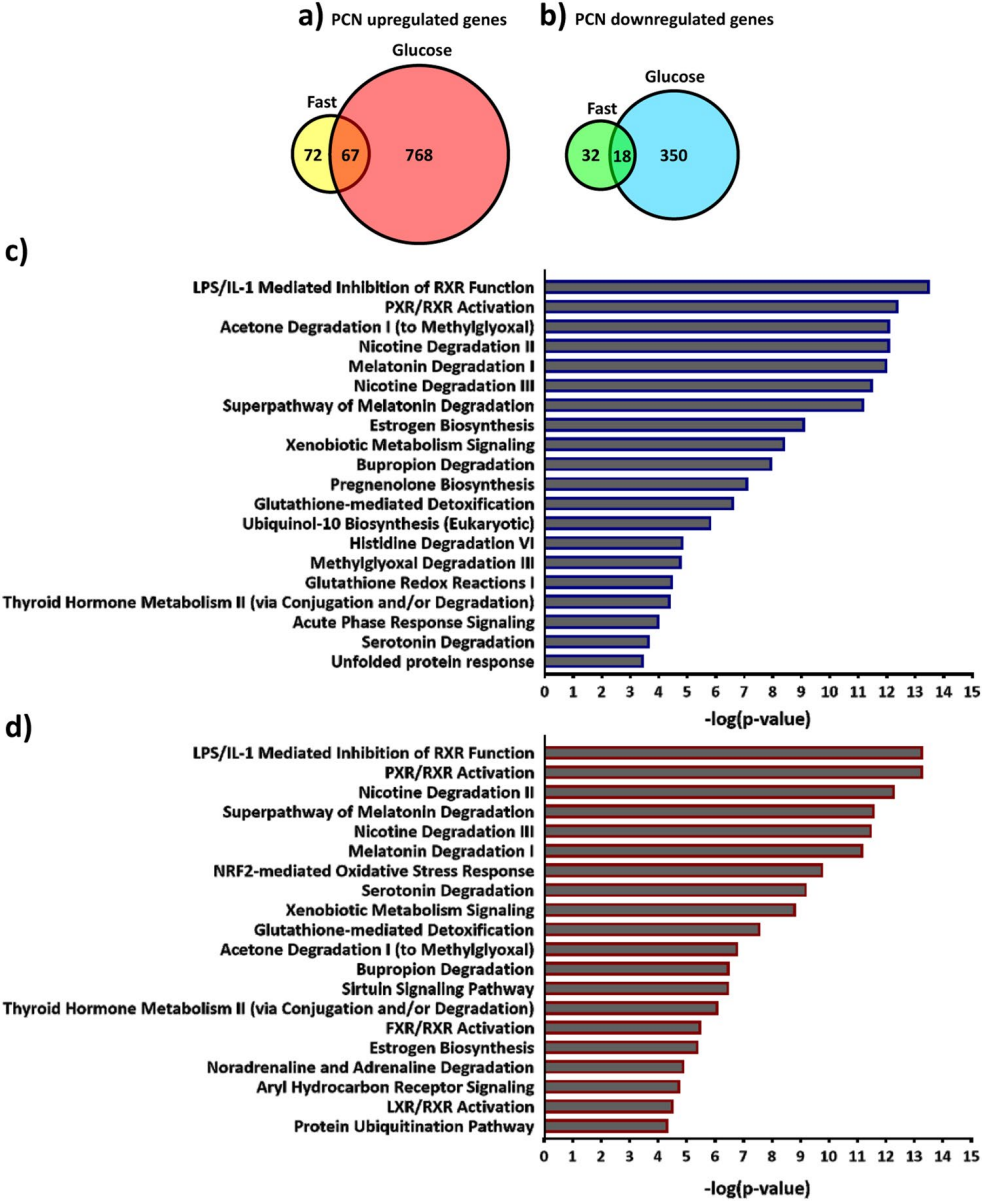
Effect of nutritional status on PCN regulated transcriptome. (**a**) Genes upregulated by PCN in the fasting and the glucose fed mice livers (threshold 1.5-fold). (**b**) Genes downregulated by PCN in the fasting and the glucose fed mice livers (threshold 1.5-fold). (**c**) The top 20 most significant canonical pathways associated with PCN-regulated gene sets in the fasting mice (negative log of P-value calculated using Fisher’s exact test). (**d**) The top 20 most significant canonical pathways associated with PCN-regulated gene sets in the glucose fed mice (negative log of P-value calculated using Fisher’s exact test).

PCN induced more genes after glucose treatment than during fasting. During fasting 139 genes were induced at least 1.5-fold and 30 genes at least 2-fold, while after glucose treatment 835 genes were induced at least 1.5-fold and 182 genes were induced at least 2-fold (Fig. 1a and Supplementary Table S1). Among the PCN upregulated genes (genes upregulated at least 1.5-fold), only 67 were common between the fasting and the glucose treatment conditions indicating significant influence of the nutritional status on PXR target gene preference (Fig. 1a and Supplementary Table S1). PCN also repressed more genes in the livers of the glucose fed mice than in the fasting mice. During fasting only 50 genes were repressed at least 1.5-fold, while after glucose treatment 368 genes were repressed to a similar degree (Fig. 1b and Supplementary Table S2). 18 genes were repressed at least 1.5-fold both in the fasting and after the glucose treatment (Fig. 1b and Supplementary Table S2).

To explore the differences in the PCN mediated gene regulation during fasting and after glucose feeding we performed Ingenuity pathway analysis (IPA). In the fasted mice the top canonical pathways affected (based on P-value < 0.05 (i.e., −log10 ≥ 1.3), Fisher’s exact test) were mainly related to metabolism of xenobiotics as well as several metabolic pathways catalyzing synthesis or catabolism of hormones or other endobiotics (Fig. 1c). Typical for these pathways is the involvement of cytochrome P450 (CYP) enzymes. In addition, some inflammation related pathways such as “LPS/IL-1 mediated inhibition of RXR function” and “Acute phase response signaling” are among the top 20 canonical pathways (Fig. 1c).

The top 20 pathways were quite similar also in the glucose fed mice (Fig. 1d). However, the top pathways included also lipid and bile acid related pathways such as “FXR/RXR Activation” and “LXR/RXR Activation” and a pathway related to energy homeostasis: “Sirtuin signaling Pathway”. Furthermore, “NRF2-mediated Oxidative Stress Response” pathway was significantly affected suggesting increase in the cellular oxidative stress.

**Figure 2:**
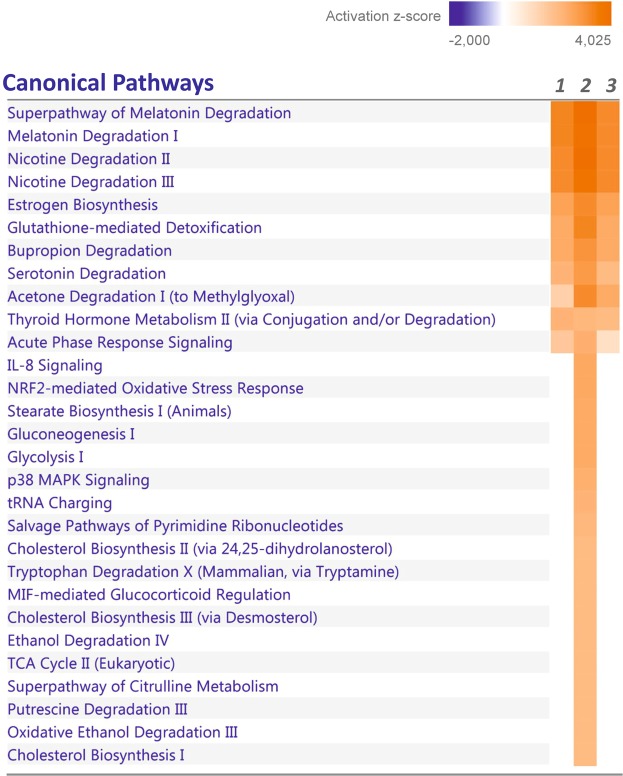
Effect of PCN treatment on predicted activation of the canonical pathways in liver based on z-score calculation by IPA software (z-score ≥2 or ≤−2). Lane 1, fasting mice; lane 2, glucose fed mice; lane 3, analysis of the gene set regulated by PCN both in fasting and in the glucose fed mice.

To further evaluate the effect of the nutritional status on PCN prompted canonical pathways we compared the activation z-scores between fasting and glucose fed mice (Fig. 2). This analysis revealed clearly that PCN activates many more functional pathways in the glucose fed mice livers than in the fasting mice. Furthermore, several of these additional pathways (“Glycolysis I”, “Gluconeogenesis I”, “Stearate biosynthesis” and “Cholesterol biosynthesis” (several different)) are related to glucose and lipid metabolism (Fig. 2, Supplementary Table S3). Although the gene expression data suggest activation of cholesterol synthesis, the plasma cholesterol level was not affected by PCN either in the fasting or the glucose fed mice (Supplementary Fig. S1).

We also used IPA to predict upstream regulators involved. Expectedly, PXR (NR1I2) and a close related nuclear receptor CAR (NR1I3) are among the predicted top upstream regulators according to the z-score. However, in the glucose fed samples Nuclear factor (erythroid-derived 2)-like 2 (NFE2L2, also called NRF2), a transcription factor activated by oxidative stress, is predicted to have even a higher z-score (Supplementary Fig. S2). Several regulators of energy homeostasis, such as Peroxisome proliferator activated receptor alpha (PPARA) and Sirtuin 1 (SIRT1) had predicted negative z-score suggesting repression of their function by PCN treatment. This effect was more pronounced in the samples from the glucose fed mice (Supplementary Fig. S2).

### Nutritional status modifies PCN mediated gene regulation in a target gene specific manner

**Figure 3:**
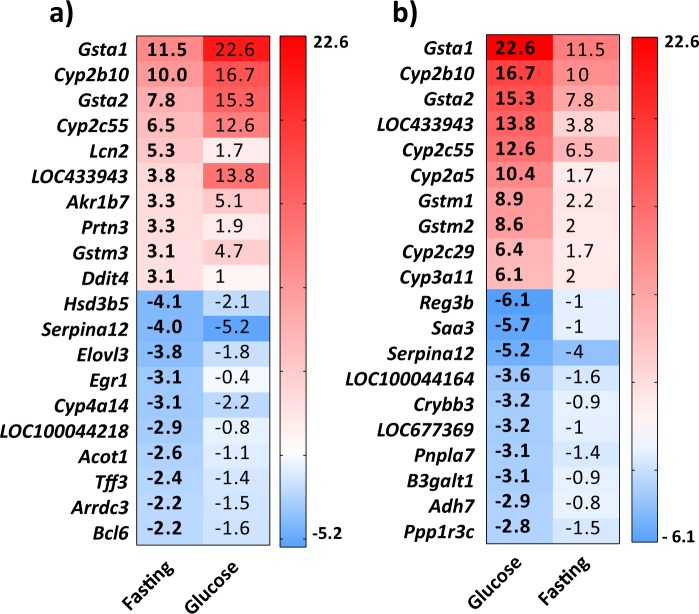
Glucose feeding affects regulation of individual genes by PCN in the liver. (**a**) Fold change of the top 10 up- and downregulated genes by PCN in the fasting mice and the corresponding fold change in the glucose fed mice. (**b**) Fold change of the top 10 up- and downregulated genes by PCN in the glucose fed mice and the corresponding fold change in the fasting mice.

Analysis of the top PCN-upregulated genes in the fasting mice indicated the expected profile of many genes coding for drug metabolizing enzymes (Fig. 3a). Interestingly, many of the top upregulated genes in the fasting mice were induced much more potently by the PCN treatment in the mice treated with glucose (Fig. 3a). The top four upregulated genes were all induced 1.5 to 2 times more potently in the livers of the glucose treated mice compared to the fasting mice. We also analyzed the top PCN-upregulated genes in the glucose treated mice. The list of the top 10 upregulated genes is rather similar to the fasting animals and include phase 1 and phase 2 drug metabolizing enzymes, however, the fold changes are all higher in the glucose fed mice (Fig. 3b).

Since the glucose feeding appeared to modify the PCN response of individual genes, we next filtered the gene list to find the genes, for which the PCN response was the most strongly affected by the glucose feeding. The top 20 genes with the highest ratio of the PCN induction fold in the glucose fed and the fasting mice are presented in Table 1. The genes with the highest ratio contained a few genes that were clearly induced by PCN in both conditions such as *Cyp3a11* and *Gstm2*; however, many genes in this list are either nonresponding or repressed during fasting but induced after glucose treatment. The gene with the highest ratio was *Cyp8b1*, one of the key genes in the bile acid synthesis. *Cyp8b1* was repressed about 50% in the fasting mice but induced almost 5-fold in the glucose fed mice (Table 1).

**Table 1:** The top 20 induction ratios for genes regulated by PCN between the fasting and the glucose fed mice livers.

Gene Symbol	Relative PCN effect		
	Fasting	Glucose	Ratio (Glucose/Fasting)
1. *Cyp8b1*	0.5	4.9	**9.8**
2. *Egr1*	0.3	2.6	**8.0**
3. *Cyp2a5*	1.7	10.4	**6.3**
4. *Gstm2*	2.0	8.6	**4.3**
5. *Cyp4a12a*	0.6	2.4	**4.2**
6. *Ephx1*	1.3	5.5	**4.1**
7. *Gstm1*	2.2	8.9	**4.1**
8. *Cyp4a12b*	0.5	1.8	**4.0**
9. *Ces3*	1.0	4.0	**3.9**
10. *Cyp2c29*	1.7	6.4	**3.8**
11. *Osgin1*	0.6	2.2	**3.7**
12. *LOC433943*	3.8	13.8	**3.7**
13. *LOC100044218*	0.3	1.2	**3.6**
14. *Es31*	0.8	2.5	**3.3**
15. *Cyp3a11*	2.0	6.1	**3.1**
16. *8430408G22Rik*	0.9	2.8	**3.1**
17. *Plec1*	1.0	3.0	**3.0**
18. *Cyp2c37*	0.8	2.3	**3.0**
19. *Cyp2f2*	1.0	2.9	**2.9**
20. *Gadd45a*	1.1	3.2	**2.9**

To determinate the effect of glucose feeding on PXR function more precisely, selected genes with different response types in the gene expression profiling analysis were further investigated with QPCR using larger animal groups. Three categories were identified: (1) genes, for which the PCN induction was potentiated by PCN, (2) genes, which were induced similarly by PCN after fasting and glucose treatment, (3) genes regulated in opposite directions by PCN after fasting and glucose feeding.

**Figure 4:**
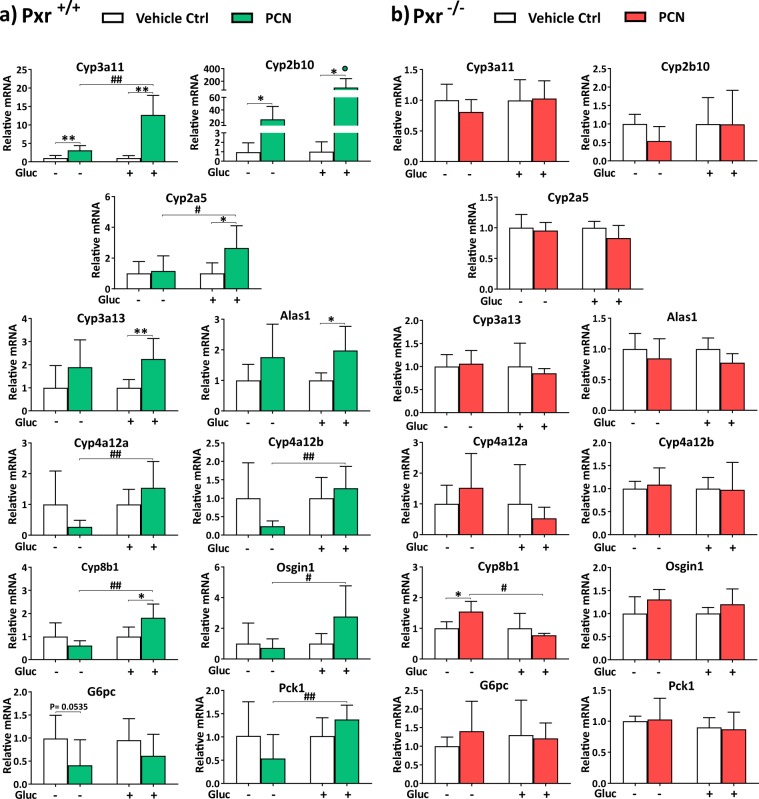
Effect of PCN treatment on selected genes in the livers of fasting and glucose fed (**a**) wildtype mice (Pxr^+/+^) and (**b**) PXR knockout mice (Pxr^−/−^). Values are presented as means ± SD. * or ^#^P < 0.05, ** or ^##^P < 0.01. In the Fig. 4a, Cyp2b10 panel one outlier value was excluded from the calculations but the value has been indicated as a green dot.

According to the microarray analysis, *Cyp3a11*, *Cyp2b10* and *Cyp2a5* were all induced by PCN during fasting, but glucose potentiated the PCN effect (Fig. 2.). Indeed, in the QPCR analysis, *Cyp3a11* and *Cyp2b10* were both induced by PCN in the fasting condition by 3- and 25-fold, respectively. However, both genes were induced much more potently after glucose feeding. *Cyp3a11* was induced 13-fold after glucose treatment, i.e. glucose potentiated the PCN mediated induction about four-fold (P < 0.01) (Fig. 4a). Also, for *Cyp2b10*, the glucose treatment quadrupled the PCN response; however, the difference in the PCN induction between the glucose fed mice and the fasting mice was not statistically significant (Fig. 4a). In contrast with the gene expression array result, the *Cyp2a5* expression was not affected by PCN in the fasting condition when measured with QPCR in larger animal groups. However, after glucose feeding PCN induced *Cyp2a5* expression 2.7-fold (Fig. 4a).

According to the gene expression profiling analysis, the PCN mediated induction of *Cyp3a13* and 5’-aminolevulinate synthase 1 (*Alas1*) was not affected by the nutritional state (Supplementary Table S1). This was supported also by the QPCR analysis of the bigger sample size. Both genes were induced by PCN about 2-fold both during fasting and after glucose feeding. However, the PCN response was statistically significant only after glucose, apparently because of the smaller variation in the mice treated with glucose (Fig. 4a).

*Cyp8b1*, *Cyp4a12a*, *Cyp4a12b* and oxidative stress induced growth inhibitor 1 (*Osgin1*) were among a third group of genes. In the gene expression array, they were all repressed in the fasted mice (Supplementary Table S2). However, they were induced in the livers of the glucose treated mice and were among the genes for which the PCN response was the most affected by the glucose administration (Table 1, Supplementary Table S1). In the QPCR analysis, they appeared to be repressed by PCN during fasting, although this decrease was not statistically significant. During glucose feeding *Cyp8b1* was significantly induced by PCN (P < 0.05), while for the other genes there was a trend for upregulation, but no statistically significant effect. Nevertheless, for all these genes, glucose feeding significantly changed the PCN response from downregulation to upregulation (Fig. 4a).

As a control, we measured expression of two glucose metabolism related genes previously reported to be repressed by PXR activation^5,6^. As expected, both glucose-6-phosphatase (*G6pc*) and phosphoenolpyruvate carboxykinase (*Pck1*) tended to be repressed by PCN in the fasting mice (Fig. 4a). Glucose treatment had significant effect on *Pck1* response to PCN, but not on that of *G6pc* (Fig. 4a). Altogether, the QPCR results confirmed the overall gene expression patterns observed in the gene expression profiling analysis and indicate that nutritional status modifies PCN mediated gene regulation in a target gene specific manner.

### PXR is indispensable for the PCN induced gene regulation and the nutritional status dependent modifications

To establish the involvement of PXR in the observed gene regulation phenomena by PCN and glucose feeding we utilized PXR knockout mice. The *Pxr*^−/−^ mice were subjected to identical experiment with four-day PCN treatment and subsequent fasting or glucose feeding as describe above. The PXR knockout abolished all the regulatory effects observed in the wildtype mice indicating that PXR is crucial for PCN mediated gene regulation as well as for the glucose induced alteration of the responses (Fig. 4b).

### Effect of glucose, insulin and glucagon on PXR mediated target gene response in mouse primary hepatocytes

In the current animal experiment, we used glucose feeding as the nutritional model. We therefore hypothesized that the increased postprandial glucose level could have a direct effect on PXR mediated gene regulation. To test this hypothesis, we utilized cultured mouse primary hepatocytes and investigated the effect of glucose concentration on PXR mediated gene regulation.

**Figure 5:**
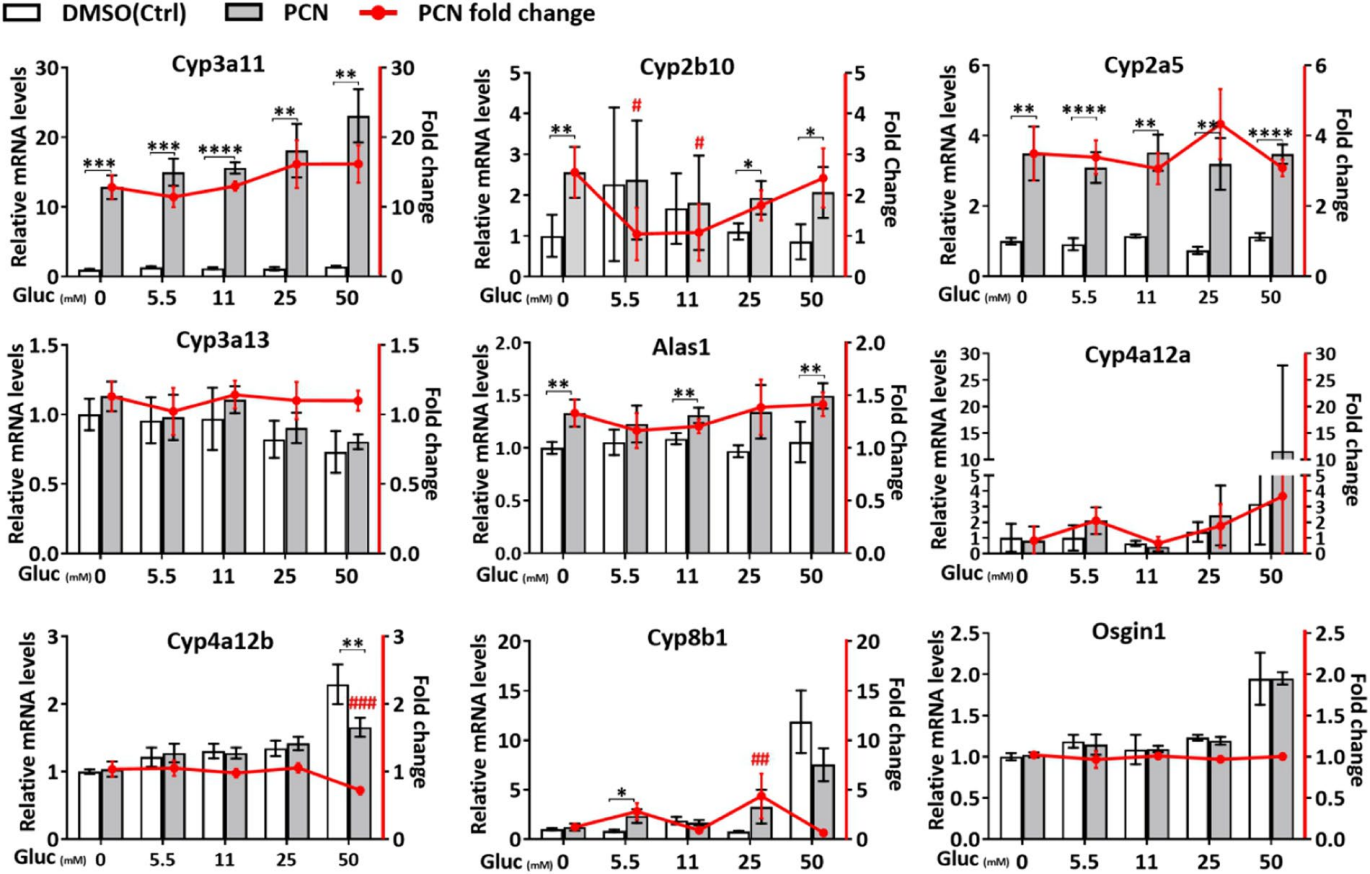
Effect of glucose (Gluc) concentration on PCN mediated gene regulation in mouse primary hepatocytes. The left axis and the columns indicate relative mRNA expression, while the right axis and the red line indicate the fold induction. Values are presented as means ± SD. * or ^#^P < 0.05, ** or ^##^P < 0.01, *** or ^###^P < 0.001, ****P < 0.0001.

*Cyp3a11* was induced efficiently by PCN in the cultured hepatocytes, and the induction magnitude was comparable to the one in the liver *in vivo*. In contrast, some other genes like *Cyp2b10* were induced poorly by PCN in the cultured hepatocytes compared with the liver *in vivo* (Fig. 5). High glucose level tended to increase *Cyp3a11* induction by PCN, from 13-fold induction with in the cells with no glucose to 16-fold induction in the cells with 25 and 50 mM glucose in the media; however, the effect was rather modest and was not statistically significant (Fig. 5). The glucose itself did not influence *Cyp3a11* expression (Fig. 5). We also tested the effect of glucose feeding alone on *Cyp3a11* expression in the liver in a separate experiment in mice. However, there was no effect (Supplementary Fig. S3). For the other genes studied, glucose level did not have any consistent, dose responsive effect on the PCN mediated regulation in the mouse primary hepatocytes. These results suggest that glucose level itself does not play a major role in the observed feeding effect on PXR mediated gene regulation in the liver.

**Figure 6:**
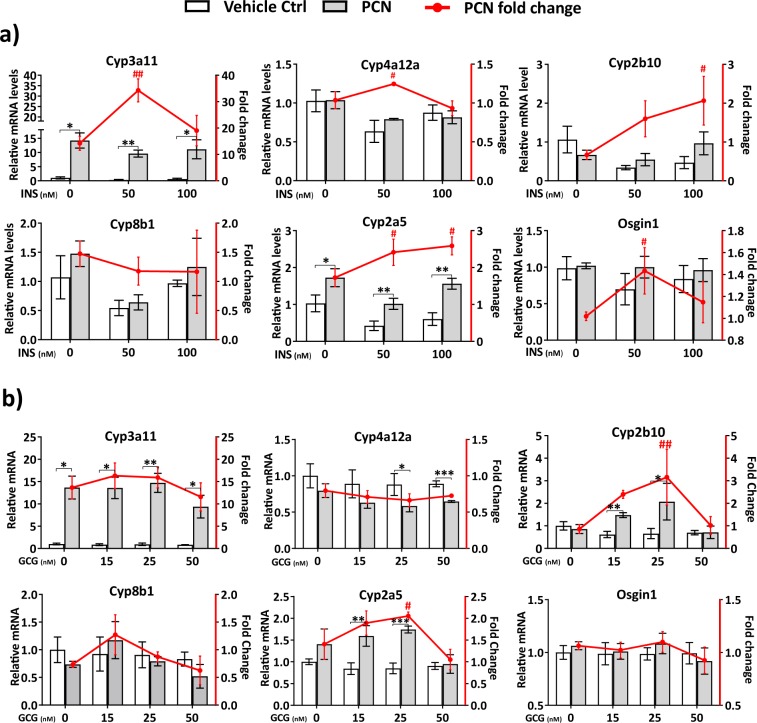
Effect of (**a**) insulin (INS) and (**b**) glucagon (GCG) on PCN mediated gene regulation in mouse primary hepatocytes. The left axis and the columns indicate relative mRNA expression, while the right axis and the red line indicate the fold induction. Values are presented as means ± SD. * or ^#^P < 0.05, ** or ^##^P < 0.01, ***P < 0.001.

Feeding induces multiple systemic effects that could modify PXR function. Fasting to feeding transition greatly changes balance of glucagon and insulin, two hormones with major effects on liver functions. We therefore studied the effect of these pancreatic hormones on PCN mediated gene regulation in primary hepatocytes. Insulin treatment increased *Cyp2a5* and *Cyp2b10* induction dose dependently (Fig. 6). Cyp3a11 induction was increased 2.4-fold with 50 nM insulin compared to no insulin in the culture media (Fig. 6). However, further upregulation of insulin had negative effect on induction.

Glucagon had the clearest effect on *Cyp2b10* induction that was dose dependently potentiated until 25 nM concentration (Fig. 6). Also, *Cyp2a5* induction was increased by 25 nM glucagon (Fig. 6). Otherwise, glucagon had no effect on PCN mediated induction of the studied genes.

### Effect of PCN on bile acid profile in glucose treated mice

Glucose feeding had dramatic effect on regulation of *Cyp8b1* by PXR (Fig. 4a, Table 1). While PCN repressed *Cyp8b1* in the fasting mice, the effect was transformed into induction after glucose feeding. Regulation of other genes coding for the key enzymes and transporters involved in liver bile acid homeostasis was explored based on the gene expression array data. *Cyp7a1* and *Cyp7b1* catalyzing key steps in the initiation of the classical and alternative bile acid synthesis pathways were systematically repressed both during fasting and after glucose treatment, although the *Cyp7a1* repression during the fasting and *Cyp7b1* repression after glucose did not reach the 1.5-fold threshold (Supplementary Table S4. *Cyp27a1* was induced only after glucose treatment (Supplementary Table S4). Among the bile acid transporters, ATP binding cassette subfamily C member 3 (*Abcc3*) was induced both during fasting and after glucose, however, the induction was about 2 times more powerful after glucose treatment. Abcc2 was induced only after glucose treatment (Supplementary Table S4). Altogether these data indicate that nutritional status modifies the response of the bile acid homeostasis related genes to PCN. Nevertheless, the most prominent change is the reversed regulation of the *Cyp8b1* during fasting and after glucose feeding.

**Figure 7:**
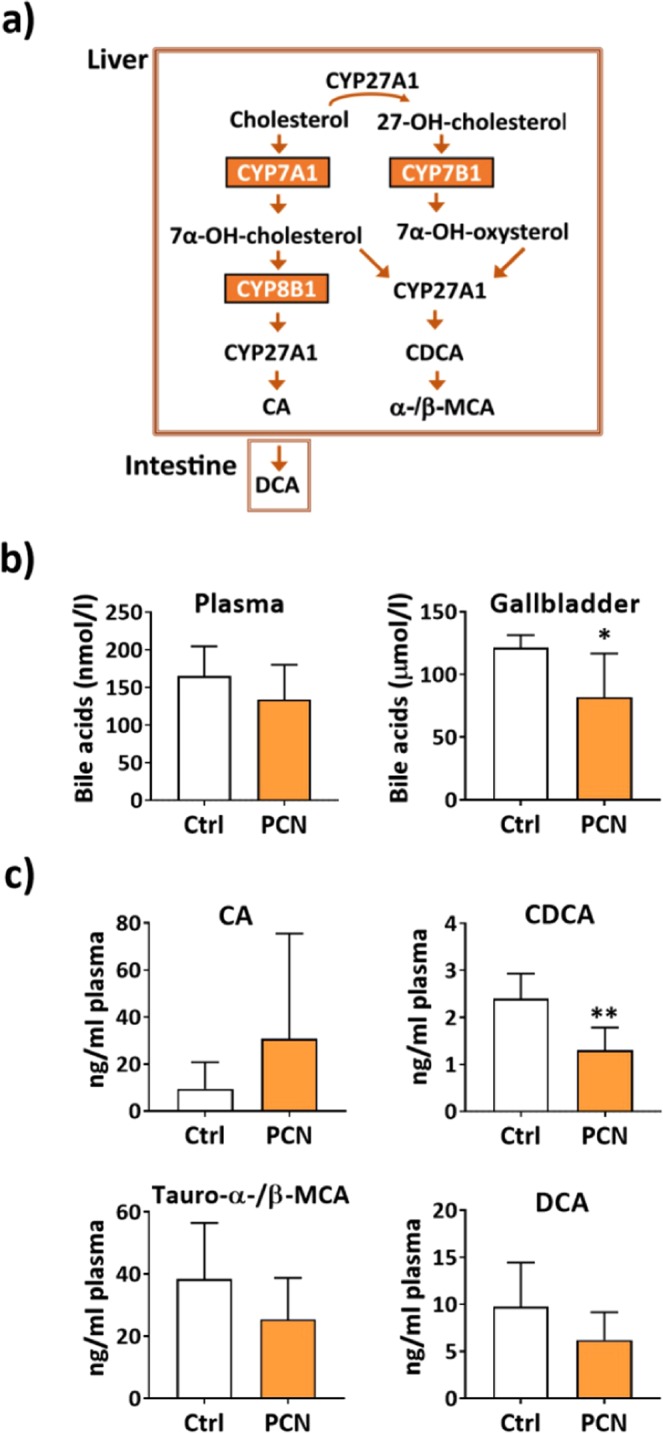
Effect of PCN treatment on bile acid synthesis in glucose fed mice. (**a**) A schematic presentation of the bile acid synthesis. Only the major steps are presented. CYP7A1 represent a rate-limiting step in the classical pathway. CYP8B1 is a branch enzyme the directs synthesis to CA. In mice the CDCA is further metabolized to α- and β- MCA. DCA is formed by the intestinal bacteria. (**b**) Total bile acid level in the plasma and in the gallbladder bile. (**c**) Levels of individual bile acids in the plasma. Values are presented as means ± SD. *P < 0.05, **P < 0.01.

CYP8B1 enzyme plays a major role in determination of the bile acid composition and functions as the branch enzyme directing synthesis towards cholic acid (CA) (Fig. 7a). Therefore, increased *Cyp8b1* expression could be expected to increase synthesis of CA and decrease production of chenodeoxycholic acid (CDCA), which mice, unlike humans, further metabolize to α- and β-muricholic acid (MCA) (Fig. 7a).

Plasma and gallbladder total bile acid levels were measured from mice treated with PCN for four days, fasted overnight and then were given glucose (2 g/kg) by oral gavage 1 h before sacrificing. There was a tendency for lower plasma levels of the total bile acids, however, the difference was not statistically significant. Instead, the gallbladder bile acids were significantly reduced by PCN supporting the finding that PCN treatment suppresses bile acid synthesis through repression of the *Cyp7* enzymes (Fig. 7b). Furthermore, individual bile acids were measured from the plasma. CA plasma level was increased, however, there were large interindividual differences in the CA levels and the difference was not statistically significant. CDCA level was reduced significantly by PCN treatment (Fig. 7c). Tauro-α/β-MCA, the further metabolites of the CDCA, and the secondary bile acids deoxycholic acid (DCA), metabolized by the intestinal bacteria from CA, were also lower, however not significantly. Altogether, these results suggest that PCN treatment combined with glucose feeding suppresses the total bile acid production and directs bigger proportion of the synthesis to the CA pathway instead of the CDCA pathway.

## Discussion

Since the early reports 15 years ago associating PXR with the regulation of glucose and lipid metabolism^6,16^, mounting evidence has been connecting PXR to different aspects of hepatic energy homeostasis^3,17^. However, in many ways the mechanisms are still unclear and discrepant results have been published by different groups using various models^3^. One potential confounding factor is the influence of the nutritional status on the PXR function.

In the present study we show that nutritional status has dramatic effects on PXR mediated gene regulation. Treatment of mice with oral glucose both qualitatively and quantitatively modified the PCN mediated transcriptome in the liver in comparison to the fasting condition. In the glucose treated mice, PCN induced six times and repressed seven times more genes than in the fasting mice. Less than half of the genes induced by PCN during fasting were affected after glucose treatment indicating significant diversity in the PCN responsive transcriptome in the different nutritional conditions. The liver samples were collected 1 hour after glucose treatment with an intention to investigate the fasting to feeding transition. Although the time point is rather early, it is expected to be sufficient for transcriptional changes^18^. The early time point decreases the chance to detect indirect PXR target genes. Nevertheless, the observed changes in the transcriptome most probably reflect changes both in transcription and in transcriptome stability and dynamics.

Both in the fasting and in the glucose treated mice livers, the top upregulated genes were mainly related to the metabolism of xenobiotics. Indeed, this represents the classical and the most well-established function under the PXR control^19^. In addition, the most strongly and significantly affected canonical pathways were mostly related to xenobiotic metabolism or other cellular pathways involving cytochrome P450 enzymes. In accordance with the higher number of genes regulated by PCN after glucose treatment, in the glucose fed mice a much higher number of canonical pathways were activated by PCN. Interestingly, many of these additional pathways were predominantly related to the glucose and lipid metabolism. Indeed, PXR is known to affect the glucose and lipid metabolism in liver^3^. These findings suggest that PXR function is adapting in the postprandial phase to the needs of the nutritional status.

In addition to the differences in the number and the type of genes affected by PCN in the different nutritional conditions, glucose treatment also modified the response of individual PXR target genes to the PCN treatment. At least three different categories of PXR target genes could be identified. Some genes, such as *Cyp3a13* and *Alas1*, were induced similarly both in the fasted and the glucose fed animals. The second group, including the prototypic PXR target gene *Cyp3a11*, was induced in both conditions, however, the glucose feeding potentiated the induction. Third, for some PCN regulated genes the glucose treatment appeared to change the direction of the response. This response type included *Cyp8b1* coding for one of the key bile acid synthesis genes. Experiment in PXR knockout mice confirmed that all these changes require PXR.

In agreement with the previous results, PXR activation by PCN decreased biliary bile acid level^20^. This is probably related to the repression of *Cyp7a1* controlling the major bile acid synthesis pathway and, to lesser extent, repression of *Cyp7b1* controlling the alternative bile acid synthesis pathway^21^. Furthermore, the sinusoidal efflux transporter *Abcc3* was also induced which may facilitate bile acid transfer to plasma. The repression of *Cyp7a1* and the induction of *Abcc3* by PXR activation are in agreement with the previous studies^20,22,23^.

Curiously, the glucose feeding had a very strong effect on regulation of *Cyp8b1*. In the fasting mice, PCN tended to repress *Cyp8b1* expression in agreement with previous studies^16,24^. However, in the glucose fed animals *Cyp8b1* was induced. CYP8B1 is a branch enzyme directing bile acids synthesis towards cholic acid (CA) and therefore the higher CYP8B1 enzyme expression is expected to have opposite effects on CA and CDCA synthesis (Fig. 7a). Indeed, PCN treatment of the glucose fed mice decreased the CDCA level significantly. Furthermore, the CA level tended to be higher. These results suggest that, in addition to the total bile acid level, PXR activation in the postprandial state may affect the bile acid composition.

Bile acids function as part of the digestive system facilitating intestinal absorption of dietary fats and oils. Moreover, bile acids are important signaling molecules activating several cellular receptors, especially the nuclear farnesoid X receptor (FXR) and the takeda G protein-coupled receptor 5 (TGR5) and regulate many important, physiological functions such as carbohydrate and lipid metabolism and immune system^25^. Individual bile acids have different potencies to activate bile acid receptors. For example, FXR is activated by CDCA > DCA > CA, while T-α- and T-β-MCA are antagonists^25^. Thus, modulation of bile acid composition may significantly affect the bile acid regulated functions. Indeed, PXR is known to be involved in regulation of glucose and lipid homeostasis and inflammation, all functions affected by bile acids^3,4^.

Our current study indicates that nutritional status has a major modifying effect on PXR mediated gene regulation. However, the exact mechanisms involved remain to be determined in the future studies. A recent study reported that high-glucose induces *CYP3A4* mRNA level and PXR regulated reporter gene activity in HepG2 cells^26^. *CYP3A4* mRNA was induced by high-glucose also in the human intestinal LS180 cells, but very modestly and not significantly in the human primary hepatocytes^26^. Furthermore, glucose level has been reported to regulate PXR phosphorylation status^15^. In the present study, we did not observe any effect of glucose alone on *Cyp3a11* expression in the mouse primary hepatocytes with concentrations up to 50 mM. Furthermore, glucose feeding alone did not influence *Cyp3a11* expression in the mouse liver. 25–50 mM glucose potentiated slightly, but no significantly, PCN mediated induction of *Cyp3a11* in cultured hepatocytes. For the other genes studied, glucose did not have any consistent effect on PCN response. These results suggest that postprandial increase in plasma glucose level is not sufficient to mediate directly the observed changes in the PXR function by glucose feeding.

Moreover, we studied the effects of insulin and glucagon on PCN induced gene regulation in primary hepatocytes. The most consistent effect was the ability of both insulin and glucagon to stimulate the induction of *Cyp2a5* and *Cyp2b10* by PCN. The mechanisms of these effects are currently unknown. However, insulin and glucagon regulate many hepatic transcription factors, such as FOXO1, SREPBP1 and PGC-1α, that are known to interact with PXR protein^6,12,16,27^.

In summary, our study provides a genome wide view to the effect of nutritional status on PXR mediated gene regulation. In response to glucose feeding the PXR regulates, directly or indirectly, much higher number and wider range of genes than during fasting. Many well-established PXR target genes are induced more powerfully by a PXR ligand after glucose feeding that during fasting, while some are regulated in a totally opposite manner during fasting and glucose feeding. These results indicate that nutritional status should be carefully controlled and reported in studies investigating PXR mediated gene regulation. Furthermore, these results provide further insight to the crosstalk of PXR and energy homeostasis.

## Methods

### Animal experiments

Eight-week-old wildtype C57BL/6 mice or *Pxr*^−/−^ mice in C57BL/6 background were used in this study. The *Pxr*^−/−^ mouse line was kindly provided by Dr. Wen Xie (University of Pittsburgh). The mice were treated *i.p*. with pregnenolone-16-α-carbonitrile (PCN, 50 mg/kg dissolved in DMSO-corn oil 1:3) or vehicle (DMSO-corn oil 1:3) once daily for four days. The mice were fasted for five hours before sacrificing or they were first fasted for four hours and then administered glucose (2 g/kg) by oral gavage one hour before sacrificing. The livers were collected and frozen in liquid nitrogen and used for total RNA extraction. The number of wildtype mice was 8 in the vehicle treated fasting group and 7 in all the other groups. Three randomly selected samples from each group was used for gene expression profiling with microarray, while all the samples were used for the QPCR measurements. The number of Pxr^−/−^ mice was 4 in each group. Furthermore, the same experimental setting was applied to the mice used for bile acid measurement with the exception that the fasting time was 12 hours before the 1-hour glucose treatment (6 mice per group).

Furthermore, the effect of glucose alone was studied in a separate experiment. C57BL/6 mice were fasted for five hours before sacrificing or they were first fasted for four hours and then administered glucose (2 g/kg) by oral gavage one hour before sacrificing. The livers were collected and frozen in liquid nitrogen and used for total RNA extraction. The number of mice was 7 in the both groups. The animal experiments were approved by the National Animal Experiment Board in Finland (license numbers ESAVI/6357/04.10.07/2014 and ESAVI/8240/04.10.07/2017) and were performed according to the EU legislation (Directive 2010/63/EU).

### Gene expression profiling

Total RNA from mouse liver tissue was isolated by using hybrid RNAzol® RT (Sigma Aldrich, St. Louis, MO, USA)/RNeasy kit (Qiagen, The Netherlands) protocol. Briefly, the tissue was homogenized into RNAzol RT solution and after mixing with RNase free water, the RNA containing supernatant was transferred to RNeasy column and the RNeasy protocol was then followed according to the manufacturer’s instructions. The quality and integrity of the isolated RNA was monitored by QIAxcel analysis according to the manufacturer’s instructions. The RNA Integrity Score (RIS) values were above 7.0 for all the samples, indicating high-quality RNA with little degradation. RNA samples (n = 3 in each group) where first labelled using the Illumina TotalPrep RNA Amplification Kit (Life Technologies, Carlsbad, CA, USA) according to the manufacturer’s instructions, with 350 ng of RNA per sample. The quality of all cRNA samples was controlled on 2100 Bioanalyzer RNA nano chips (Agilent Technologies, Santa Clara, CA, USA) and quantified using a Nanodrop 2000 spectrometer (Thermo Scientific, Waltham, MA, USA). A total of 750 ng of each sample was used in the Direct Hybridization Assay Workflow (Illumina, San Diego, CA, USA) using MouseWG-6 v2.0 Expression BeadChips (Illumina, San Diego, CA, USA) which targets >45 200 transcripts. The BeadArrays were scanned using a HiScan instrument (Illumina, San Diego, CA, USA). The work was carried out at the Core Facility of the Estonian Genome Center, University of Tartu, Estonia. Probe intensity and detection data were obtained using Illumina BeadStudio software, and further processed with GeneSpring GX 14.5 software (Agilent Technologies, Santa Clara, CA, USA). The raw expression data was quality assessed, log2-transformed, and subjected to normalization to the 75^th^ percentile and baseline transformation to median of all samples. Genes were defined as differentially expressed if the fold change was at least 1.5-fold and statistically significant (P < 0.05, One-way ANOVA and Tukey’s post hoc -test). The complete data sets are available from the NCBI’s Gene Expression Omnibus (GEO) database and gene expression profiling data comply with the MIAME (Minimum Information About a Microarray Experiment) standard. The data can be obtained from the GEO database with the accession numbers GSE125695.

### IPA pathway analysis of differentially expressed genes

Differentially expressed genes and their corresponding expression values were uploaded into the Ingenuity Pathway Analysis (IPA) software (Qiagen, Redwood City, CA, USA), and a core analysis was performed separately for every nutritional treatment by using default parameters. The following parameters were used: core analysis, reference set user-defined (i.e., only the set of differentially expressed genes by GeneSpring-software mapped to the IPA database), direct and indirect relationships included, endogenous chemicals included, confidence = experimentally observed. Significant associations of well-documented canonical pathways in the Ingenuity Pathway Knowledge database and the differentially expressed genes were analyzed by IPA-software (Fisher’s exact test, P-value < 0.05 (i.e., −log10 ≥ 1.3)). In addition, an activation z-score was generated for each functional category. The entries that had a P-value < 0.05 and an absolute z-score value 2 were considered significant. Furthermore, we identified upstream transcriptional regulators with p-value of overlap < 0.05 and the absolute activation z-score > 2.

### Cell culture

Mouse primary hepatocytes were isolated from untreated C57BL/6 mice as described previously^7^. The cells were seeded with 300 000 cells/well in 12-well plates in plating medium (William’s E (Sigma Aldrich) with 10% FBS, supplemented with dexamethasone 10 ng/ml, gentamycin 10 µg/ml and insulin-transferrin medium supplement [containing 5 mg/L insulin, 5 mg/L transferrin and 5 µg/L sodium selenite] (Sigma Aldrich) for 3 hours. After the cells were attached, the medium was changed to serum-free plating medium. The cells were first treated with pregnenolone-16-ɑ-carbonitrile (PCN, 10 µM dissolved in DMSO) or vehicle (DMSO) for 21 h. Next the cells were then treated with different concentrations of either glucose, insulin or glucagon for 24 h while the 10 µM PCN or vehicle treatment was also continued. Glucose treatment was done in serum free DMEM with 4 replicates/group. Insulin and glucagon treatments were done in plain William´s E in triplicates. The cells were harvested for RNA isolation.

### Quantitative real-time PCR

Total RNA was isolated from liver samples, or from primary hepatocytes using RNAzol® RT followed by cDNA synthesis with p(dN)_6_ primer and RevertAid transcriptase (ThermoFisher Scientific, Waltham, MA, USA). Quantitative real-time PCRs were done with FastStart Universal SYBR Green Master Mix (Roche, Basel, Switzerland) and ABI 7300 thermal cycler (Applied Biosystems, Foster City, CA, USA), or with PowerUp™ SYBR™ Green Master Mix (Applied Biosystems) and QuantStudio 5 real-time qPCR thermal cycler (ThermoFisher Scientific). Primers used for qPCR are shown in the Table 2. Primer concentrations were optimized to achieve 100% efficiency for amplification. The fluorescence values of the QPCR products were corrected with the fluorescence signals of the passive reference dye (ROX). The RNA levels of target genes were normalized against the 18 S or GAPDH control levels using the comparative CT (ΔΔCT) method.

**Table 2:** PCR primers for quantitative real-time PCR.

Gene	Forward 5′ to 3′	Reverse 3′ to 5′
Alas1	TCGCCGATGCCCATTCTTATC	GGCCCCAACTTCCATCATCT
Cyp3a11	GACAAACAAGCAGGGATGGAC	CCAAGCTGATTGCTAGGAGCA
Cyp8b1	CCTCTGGACAAGGGTTTTGTG	GCACCGTGAAGACATCCCC
Cyp2a5	GGACAAAGAGTTCCTGTCACTGCTTC	GTGTTCCACTTTCTTGGTTATGAAGTCC
Cyp4a12a	CCTCTAATGGCTGCAAGGCTA	CCAGGTGATAGAAGTCCCATCT
Cyp2b10	AAAGTCCCGTGGCAACTTC	TTGGCTCAACGACAGCAA
Cyp4a12b	GGGGAGATCAGACCCAAAAGC	ATTCGTCGGTGCTGAAACCAT
Cyp3a13	GACGATTCTTGCTTACCAGAAGG	CCGGTTTGTGAAGGTAGAGTAAC
Gsta1	TGTTGAAGAGCCATGGACAA	ATCCATGGGAGGCTTTCTCT
GAPDH	GGTCATCATCTCCGCCCC	TTCTCGTGGTTCACACCCATC
Lcn2	TGGCCCTGAGTGTCATGTG	CTCTTGTAGCTCATAGATGGTGC
Osgin1	CCTCCGGTATCTGCCTGTC	GGAAAGGTACTCTAGGTCCTGG
Socs2	AGTTCGCATTCAGACTACCTACT	TGGTACTCAATCCGCAGGTTAG
18S	CGCCGCTAGAGGTGAAATTC	CCAGTCGGCATCGTTTATGG

### Bile acid measurements

Total bile acids from plasma and gallbladder bile were measured with Mouse Total Bile Acids Assay Kit (Crystal Chem, Zaandam, Netherlands) according to the manufacturer’s protocol.

Individual bile acids were measured from plasma. To remove plasma proteins, 50 µl of EDTA-plasma was mixed with 500 µl of acetonitrile, kept for 1 h at −20 °C and centrifuged. The supernatant was dried in a vacuum and redissolved into 50 µl of 50% acetonitrile. 5 µl of the solution was analyzed by LC/MS using Aquity UPLC system (Waters, Milford, MA, USA) connected to Synapt G1 Q-TOF mass spectrometer (Waters). The eluents were A: 5 mM ammoniumacetate, 0.018% formic acid and B: 5% acetonitrile in methanol. The linear gradient was operated at 0.4 ml/min with the following program: 0 min 40% B; 10 min 100% B, 14 min 100% B, 15 min 40% B. The column, BEH C18, 1.7 µm, 2 × 100 mM (Waters), was kept at 40 °C. The mass spectrometer collected 0,2 second scans in the mass range between 50–1500 in V-mode using negative ionization, recording centroid peaks with lock mass (Leu-encephalin) correction. Quantification was calibrated with standards at 1, 5, 10, 30, 70 and 100 ng/ml. Standards were cholic acid (C1129; Sigma), chenodeoxycholic acid (C9377, Sigma), deoxycholic acid (D2510, Sigma) and tauro α-muricholic acid (C1893–000, Steraloids Inc., Newport, RI, USA).

### Cholesterol measurement

Plasma total cholesterol was measured with Cholesterol Quantitation Kit (Sigma Aldrich) according to the manufacturer’s protocol.

### Statistical analysis

The statistical data analysis was performed using GraphPad Prism Software (La Jolla, CA, USA). The comparison of means of two groups was done by Student’s two-tailed t-test, whereas multiple groups were compared by One-way ANOVA followed by Dunnett’s post hoc test. Differences were considered significant at P < 0.05.

## Supplementary information

Supplementary Figures

Supplementary tables

## Data Availability

The datasets generated during the current study are available in the Gene Expression Omnibus (GEO) repository with the Accession Number GSE125695.
