# Supplementary material for: Nutritional status modifies pregnane X receptor regulated transcriptome
Source: Sci Rep. 2019 Nov 13;9:16728. doi: 10.1038/s41598-019-53101-9 (PMC6853963; doi:10.1038/s41598-019-53101-9)

Supplementary data to article:

Nutritional status modifies pregnane X receptor regulated transcriptome

Fatemeh Hassani-Nezhad-Gashti, Outi Kummu, Mikko Karpale, Jaana Rysä and Jukka Hakkola

Legends to the supplementary Figures

Supplementary Fig. S1. The effect of PCN and glucose on mouse plasma cholesterol level.

Supplementary Fig. S2. The activation states of predicted upstream transcriptional regulators (filtered with terms ligand-dependent nuclear receptors and transcription regulators) based on the absolute activation z-score >2. Lane 1, fasting mice; lane 2, glucose fed mice; lane 3, analysis of the gene set regulated by PCN both in fasting and in the glucose fed mice.

Supplementary Fig. S3. The effect of glucose feeding on *Cyp3a11* expression in mouse liver.

Supplementary Figure S1

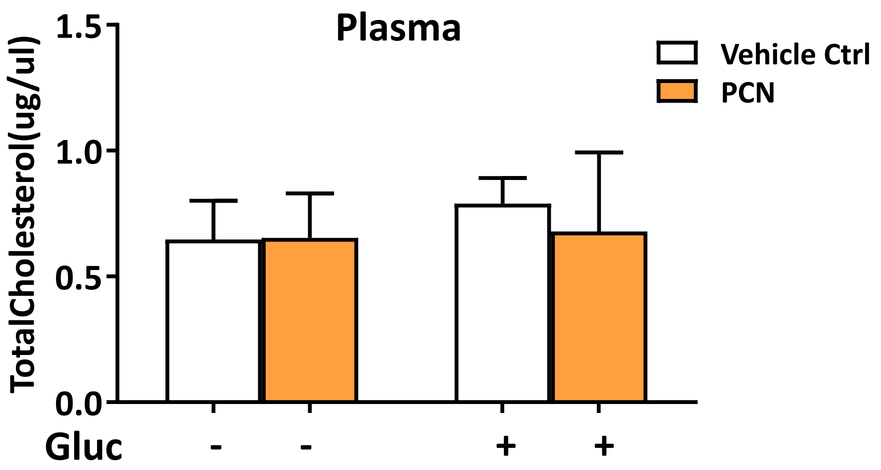

Supplementary Figure S2

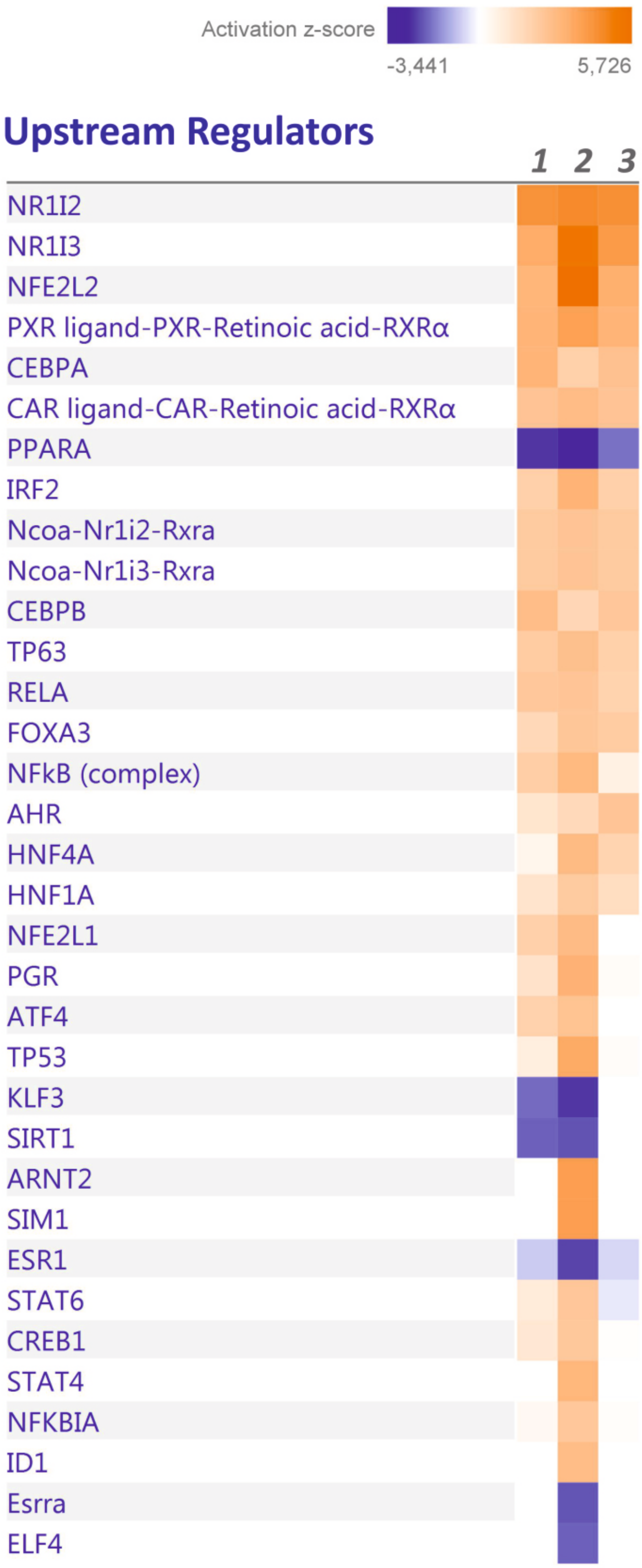

Supplementary Figure S3

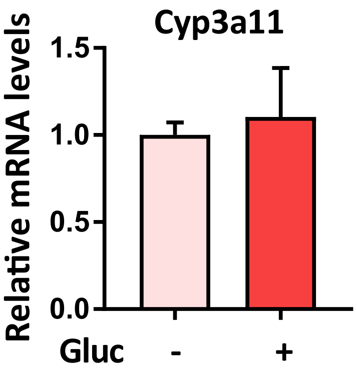

Supplement: Supplementary file 1 — Supplementary Figures [file 41598_2019_53101_MOESM1_ESM.pdf]
